# Ultrastructure and Morphology of Compound Eyes of the Scorpionfly *Panorpa dubia* (Insecta: Mecoptera: Panorpidae)

**DOI:** 10.1371/journal.pone.0156970

**Published:** 2016-06-03

**Authors:** Qing-Xiao Chen, Bao-Zhen Hua

**Affiliations:** 1 State Key Laboratory of Crop Stress Biology for Arid Areas, Entomological Museum, Northwest A&F University, Yangling, Shaanxi, China; 2 Forestry College, Henan University of Science and Technology, Luoyang, Henan, China; Lund University, SWEDEN

## Abstract

Mecoptera are unique in holometabolous insects in that their larvae have compound eyes. In the present study the cellular organisation and morphology of the compound eyes of adult individuals of the scorpionfly *Panorpa dubia* in Mecoptera were investigated by light, scanning electron, and transmission electron microscopy. The results showed that the compound eyes of adult *P*. *dubia* are of the apposition type, each eye comprising more than 1200 ommatidia. The ommatidium consists of a cornea, a crystalline cone made up of four cone cells, eight photoreceptors, two primary pigment cells, and 18 secondary pigment cells. The adult ommatidium has a fused rhabdom with eight photoreceptors. Seven photoreceptors extend from the proximal end of the crystalline cone to the basal matrix, whereas the eighth photoreceptor is shorter, extending from the middle level of the photoreceptor cluster to the basal matrix. The fused rhabdom is composed of the rhabdomeres of different photoreceptors at different levels. The adult ommatidia have the same cellular components as the larval ommatidia, but the tiering scheme is different.

## Introduction

Compound eyes are the prominent visual organs for the majority of insects [1]. Unlike the single-chamber eyes of vertebrates, the compound eyes of insects are generally composed of many independent optical units called ommatidia [2] and can perform significant functions of flight control, navigation, prey capture, predator avoidance, and mate recognition [3–6]. Due to impressive structural complexity and genetic conservation, the compound eyes play important roles in analyzing the evolution of animal eyes [7–9] and the phylogeny of Arthropoda [10,11].

The two basic optical designs of the insect compound eyes are the apposition type and the superposition type [4,12]. In the apposition type, the ommatidia are optically isolated with longitudinal pigments. In the superposition type, there is a pigmentless clear zone between the cornea and rhabdomeres [13], so that all photoreceptors share corneal dioptrical units, increasing the light sensitivity of the eye [14]. Superposition eyes are commonly found in nocturnally active insects [15–17], but also exist in some diurnally active insects [18–20]. The insect compound eyes can adapt to different environmental conditions by diverse structural modifications [21]. For many fast-flying insects such as damselflies and dragonflies of Odonata [22,23], the ommatidia located in the dorsal and ventral regions of the compound eyes have distinct morphological specializations, which may function as light detection against the sky and ground, respectively. In addition, the variations in facet diameter, interommatidial angle, photoreceptor arrangement, and rhabdom dimension allow the compound eyes to obtain more optical information under natural environments [12,21].

Compound eyes are often present in most adult insects and juvenile exopterygotes, but may be reduced or absent in some cave-dwelling, parasitic, and sedentary species [2]. Most holometabolous insects lack compound eyes in their larval stages [10]. Melzer and Paulus [24] found that the larvae of some nematoceran Diptera have compound eyes, which are nevertheless paedomorphically expressed adult compound eyes and retained until the adult stage [25]. On the contrary, the larvae of Panorpidae in Mecoptera have compound eyes that are resorbed during metamorphosis [11,26–29], unique in holometabolous insects.

Because of the possession of larval compound eyes, Mecoptera are considered one of the basal lineages in Holometabola [27,30]. The constructions of compound eyes have been investigated in the larval [29,31,32] and adult species [33–35] in Mecoptera. Ando and Suzuki [36] claimed that the compound eyes of larval Panorpidae were homologous to those of hemimetabolous insects due to similar processes of embryonic development. Melzer [37] found that some structures like lamina, medulla, and long and short visual fibers were present in both larval and adult neuropils of Panorpidae, but the lobula neuropil only existed in the adult eyes. The ultrastructure of compound eyes was recently investigated in the larval *Panorpa dubia* by Chen et al. [29]. In this paper the cellular organisation and morphology of adult compound eyes of the scorpionfly *Panorpa dubia* [38] were investigated by light, transmission electron, and scanning electron microscopy in order to compare the structure of the adult and larval compound eyes in the Mecoptera.

## Materials and Methods

### Insect Collections

Adults of *P*. *dubia* were collected by sweeping nets from 9 am to 12 am in the Zhuque National Forest Park (33°92′N, 108°52′E, elev. 1500 m), Qinling Mountains, Shaanxi Province, China from July to August in 2013.

### Light Microscopy (LM) and Transmission Electron Microscopy (TEM)

Live adults (26 males and 22 females) were anaesthetized with diethyl ether. The compound eyes were removed from the heads with a razor blade under a fluorescent lamp and immediately fixed in a mixture of 2.0% paraformaldehyde and 2.5% glutaraldehyde in phosphate buffer (PB, 0.1 M, pH 7.2) at 4°C for 6 h [39]. The fixed eyes were rinsed with PB and then post-fixed with 1% osmium tetroxide (OsO_4_) at 4°C for 2 h. The compound eyes were dehydrated through a graded series of acetone (50%, 70%, 80%, 90%, and 100%) and infiltrated successively through mixtures of acetone and Epon 812 resin (3:1, 1:1, and 1:3) and pure Epon 812 resin. The compound eyes were then embedded in Epon 812 resin with nadic methyl anhydride as hardener, dodecenyl succinic anhydride as softener, and 2,4,6-tri(dimethylaminomethyl)phenol (DMP-30) as epoxy accelerator and polymerized into the resin blocks at 30°C for 24 h and 60°C for 48 h.

For LM observations, the resin-embedded compound eyes were cut into longitudinal and transverse sections of approximately 2 μm thick on a Leica EM UC7 ultramicrotome (Leica, Nussloch, Germany). The semi-thin sections were stained with 1% toluidine blue and examined under a Nikon Eclipse 80i light microscope (Nikon Corporation, Tokyo, Japan).

For TEM observations, the resin-embedded compound eyes were cut with a diamond knife into 70 nm thick sections on the same ultramicrotome as mentioned above. The ultra-thin sections were stained with 2% uranyl acetate and 4% lead citrate for a few minutes each and observed under a JEOL JEM-1230 transmission electron microscope (JEOL, Tokyo, Japan) at 80 kV.

### Scanning Electron Microscopy (SEM)

Sample preparations for the SEM basically followed the procedure given by Chen and Hua [40]. The intact compound eyes were dissected out of the heads and fixed with Bouin’s fluid for 24 h. The compound eyes were then ultrasonically cleaned for a few seconds before dehydration in a graded series of ethanol, and passed through mixtures of ethanol and tertiary butanol and pure tertiary butanol. After freeze-drying, the eyes were separated into a number of isolated ommatidia with a razor blade under a Nikon Stereoscopic Zoom Microscope SMZ1500. These ommatidia were coated with gold and examined in a Hitachi S-3400N scanning electron microscope (Hitachi, Tokyo, Japan) at 15 kV.

Specific permits were not required as the field studies did not involve a protected species and the study locations were not privately owned or protected.

## Results

**Fig 1:**
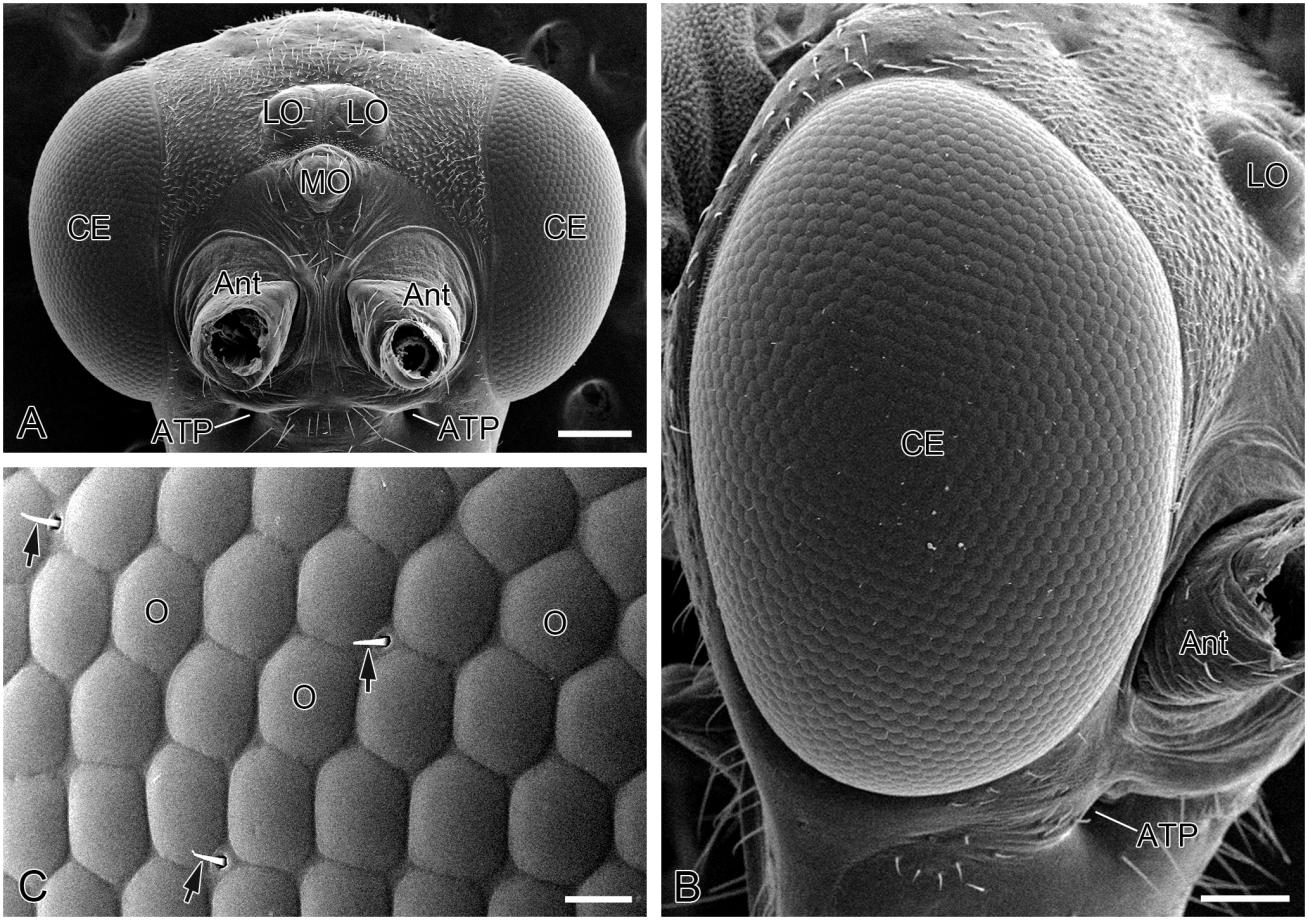
External morphology of compound eyes of adult *Panorpa dubia*, SEM. **(A)** Adult head viewed from the front, showing compound eyes on either side of the head. **(B)** Right compound eye, lateral view. **(C)** Ommatidia. Arrows indicate interfacetal hairs. Ant, antenna; ATP, anterior tentorial pit; CE, compound eye; LO, lateral ocellus; MO, median ocellus; O, ommatidium. Scale bars: (A) and (B) = 150 μm; (C) = 10 μm.

Adults of *P*. *dubia* possess a pair of elliptical compound eyes and three oval dorsal ocelli on the heads (Fig 1A). The compound eyes are blackish brown and exhibit no pseudopupils under natural conditions. Based on the light and electron microscopic observations, no sexual dimorphism and regional differences were found in the compound eyes. Therefore, we took the compound eyes of the males as representatives to uncover the construction of the compound eyes. A data collection of single specimen measurements from the presented micrographies is listed in Table 1, where the ultrastructural measurements were made from the central regions of male compound eyes of *P*. *dubia*.

**Table 1:** Measurements on the compound eyes of male *Panorpa dubia*.

Structural compositions	Morphological data	Measurements
**Compound eyes**	dorso-ventral axis length	~1.2 mm
	antero-posterior axis length	~0.8 mm
	number of ommatidia	~1200
**Ommatidia**	length	~240 μm
	facet diameter	~27 μm
**Cornea**	thickness	~18 μm
	number of chitin layers	~60
**Crystalline cone**	length	~50 μm
	distal diameter	~20 μm
**Primary pigment cells**	depth of nuclei	~60 μm
	diameter of pigment granules	~0.6 μm
**Secondary pigment cells**	depth of nuclei	~45 μm
**Photoreceptors**	cluster length	~174 μm
	starting depth of the cell cluster	~66 μm
	distal diameter of cell cluster	~10 μm
	proximal diameter of cell cluster	~6 μm
	depth of R7 tapering	~88 μm
	depth of R8 arising	~123 μm
**Rhabdom**	total length of R1–R6	~165 μm
	starting depth	~68 μm
	length of R7	~60 μm
	length of R8	~115 μm
	cross-section with R1–R7 contributing (depth of 68–123 μm)	~3.0 μm^2^
	cross-section with R1–R8 contributing (depth of 123–128 μm)	~4.6 μm^2^
	cross-section with R1–R6 and R8 contributing (depth of 128–238 μm)	~4.5 μm^2^
**Basal matrix**	thickness	~4 μm

The compound eyes of adult *P*. *dubia* measure approximately 1.2 mm along the dorso-ventral and about 0.8 mm along the antero-posterior axis and each consist of at least 1200 ommatidia (Fig 1B). All ommatidia have convex corneal lenses, which are approximately hexagonal in shape of ~27 μm in diameter (Fig 1C). Several short interfacetal hairs are present at the corners between facet lenses (Fig 1C).

**Fig 2:**
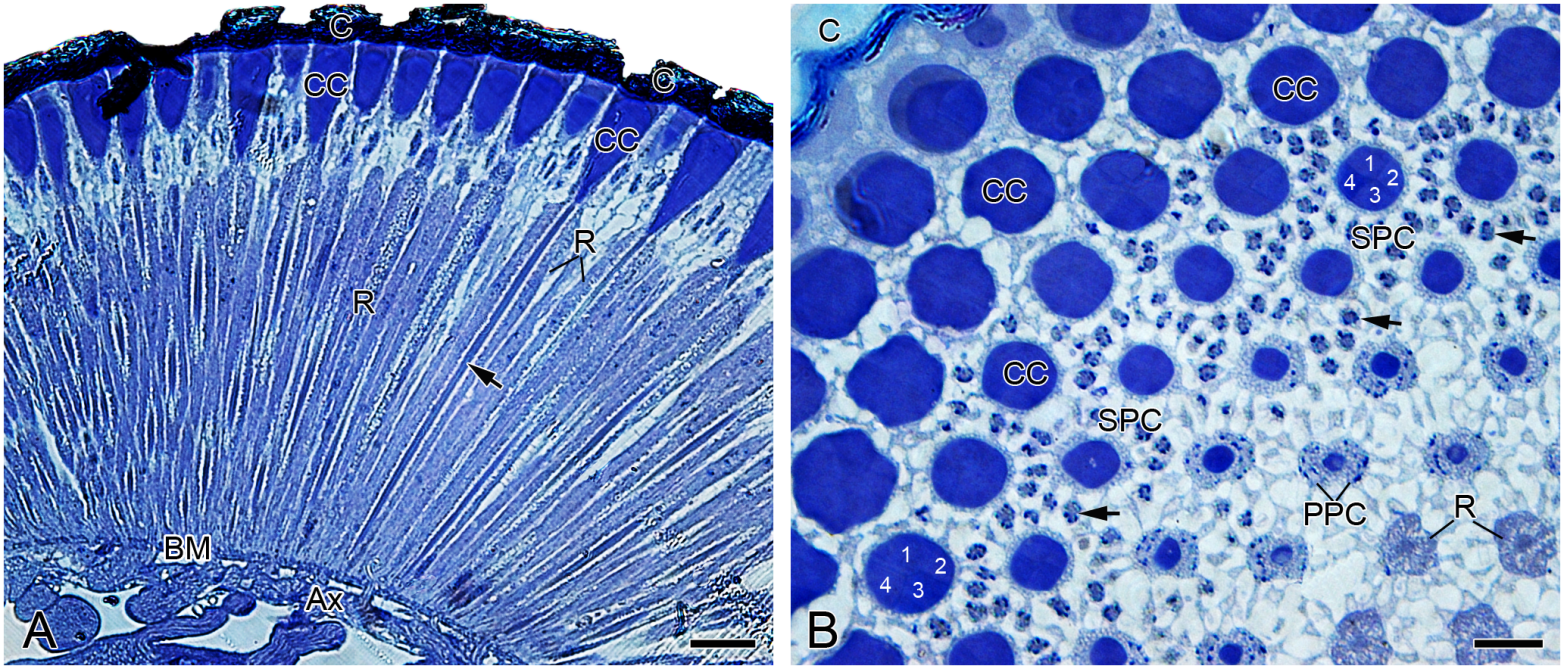
Histological sections of compound eyes of adult *Panorpa dubia*, LM. **(A)** Longitudinal section showing rhabdom (arrow) formed by photoreceptors in one ommatidium. **(B)** Transverse-oblique section of greater part of compound eye showing ommatidia cut at various levels; four cone cells are marked in one ommatidium (1–4); arrows indicate nuclei of interommatidial secondary pigment cells. Ax, axon; BM, basal matrix; C, cornea; CC, crystalline cone; PPC, primary pigment cell; R, photoreceptor; SPC, secondary pigment cell. Scale bars: (A) = 25 μm; (B) = 10 μm.

**Fig 3:**
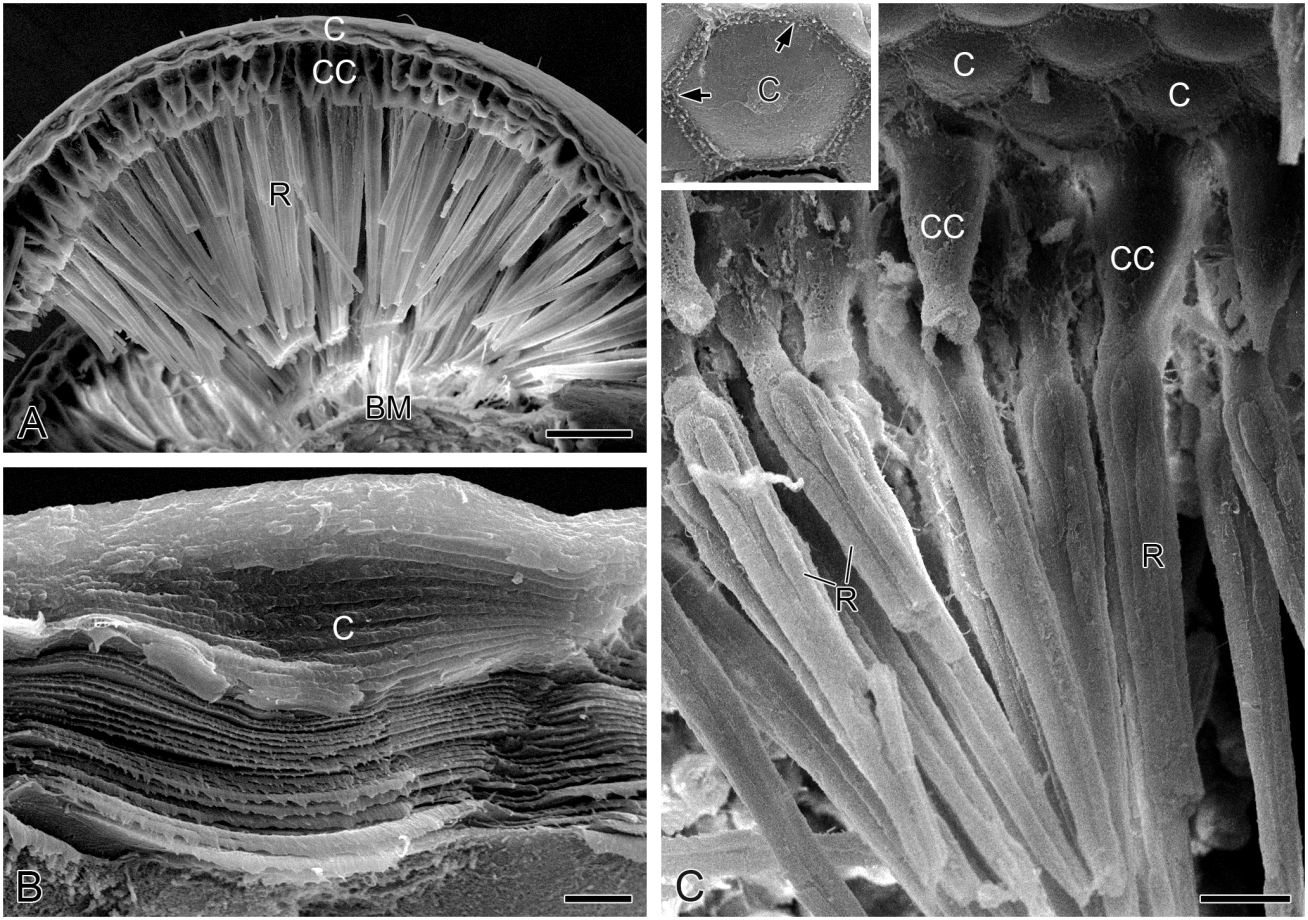
Ultramorphology of compound eyes of adult *Panorpa dubia*, SEM. **(A)** Longitudinal profile through compound eye. **(B)** Longitudinal profile through cornea showing approximately 60 lamellae. **(C)** Crystalline cones connected to rod-like photoreceptors. Inset shows that the internal surface of cornea bears numerous micropapillae (arrows) along its edge. BM, basal matrix; C, cornea; CC, crystalline cone; R, photoreceptor. Scale bars: (A) = 40 μm; (B) = 2 μm; (C) = 15 μm.

The individual ommatidium consists of a cornea, a crystalline cone, a cluster of photoreceptors, and a basal matrix (Figs 2A and 3A). The laminated cornea is about 18 μm thick and comprises approximately 60 chitin layers, which increase in thickness from proximal to distal level (Fig 3B). The internal surface of the cornea bears numerous micropapillae (Fig 3C, inset). Beneath the cornea is the crystalline cone, which is approximately 50 μm long and consists of 4 equally sized cone cells (Fig 2B). The proximal end of crystalline cone connects to the tip of photoreceptors (Fig 3C). No spacing is present between the tip of the crystalline cone and the tip of the rhabdom.

**Fig 4:**
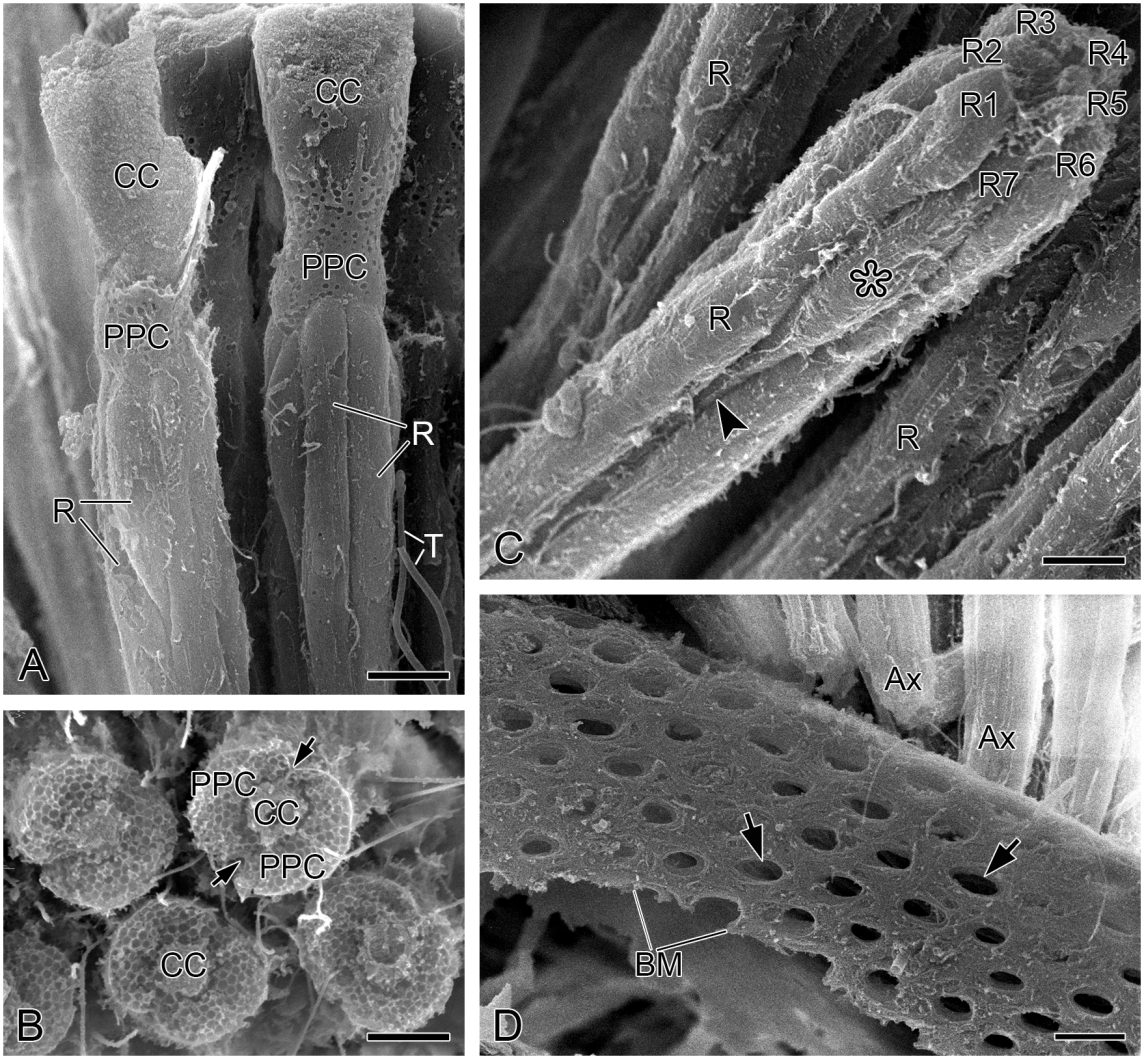
Ultramorphology of photoreceptors and basal matrix of adult *Panorpa dubia*, SEM. **(A)** Longitudinal profile through crystalline cones and photoreceptors showing that primary pigment cells surround the junction between crystalline cone and photoreceptors. **(B)** Transverse profile through two primary pigment cells. Arrows indicate the boundaries of primary pigment cells. **(C)** Seven photoreceptors (R1–R7) are identifiable. R7 has an inflated nuclear zone (asterisk) and tapers sharply toward the centre of the cluster (arrowhead). **(D)** Basal matrix, showing numerous round perforations for axon bundles to pass through (arrows). Ax, axon; BM, basal matrix; CC, crystalline cone; PPC, primary pigment cell; R, photoreceptor; T, tracheole. Scale bars: (A) and (D) = 6 μm; (B) = 5 μm; (C) = 4 μm.

A pair of primary pigment cells surrounds the crystalline cone and connects to the apical surface of photoreceptor cluster (Fig 4A). In SEM of transverse sections, numerous round pits with diameter 0.6 μm can be seen (Fig 4B). The pits are likely the remnants of pigment granules. A ring of 18 secondary pigment cells is located on the periphery of primary pigment cells and can be recognized by the locations and sizes of their respective nuclei (Fig 2B). The nuclei of primary pigment cells are at the depth of about 60 μm below the external surface of the cornea, occupying more proximal positions than those of the secondary pigment cells at about 45 μm depths (Fig 2B).

The photoreceptors of each ommatidium are arranged radially along the longitudinal axis (Fig 4C). The whole cluster of photoreceptors is about 174 μm in length and has a diameter of approximately 10 μm in the most distal level and about 6 μm in the most proximal level (Figs 2A and 3A). Eight photoreceptors are marked as R1–R8 according to the numbering system of Diptera reviewed by Friedrich et al. [41], who emphasized the developmental and structural homologies of photoreceptors as the standard of identification. Each photoreceptor possesses a stack of microvilli, which together form a rhabdomere. The fused rhabdom is about 165 μm long (Fig 2A).

**Fig 5:**
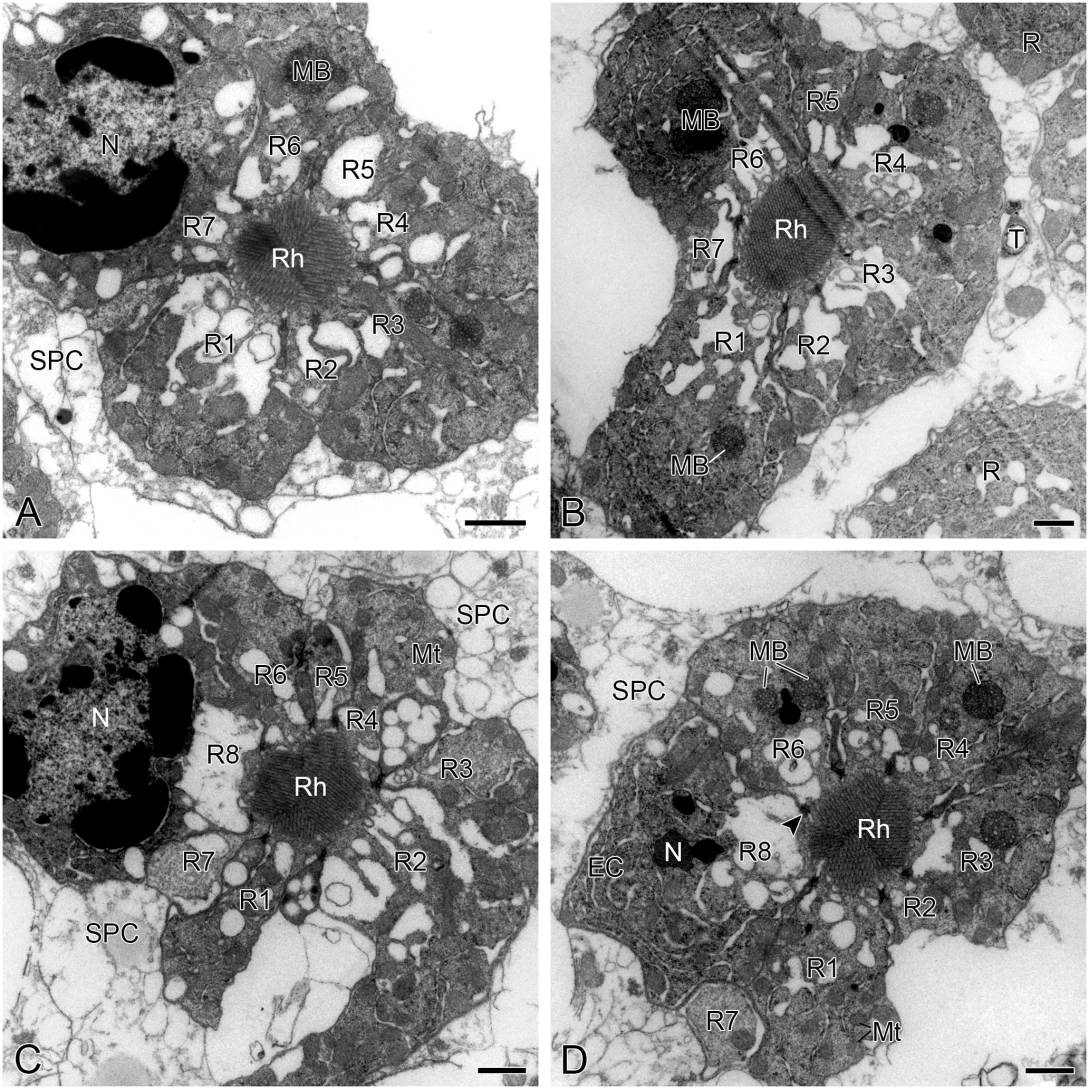
Transverse sections through rhabdom of adult *Panorpa dubia*, TEM. **(A)** The photoreceptor cluster showing that seven photoreceptors (R1–R7) form a fused rhabdom at the depth of roughly 68–88 μm. **(B)** At the depth of about 20 μm below the tips of photoreceptors R7 rapidly diminishes in size and becomes much smaller than other photoreceptors (R1–R6). **(C)** At the depth of approximately 123–128 μm the rhabdom is formed by the rhabdomeres of R1–R8. **(D)** Below about 128 μm depth the rhabdom is composed of the rhabdomeres of R1–R6 and R8. Arrowhead indicates a desmosome connecting two adjacent photoreceptors. EC, endoplasmic cistern; MB, multivesicular body; Mt, mitochondrion; N, nucleus; R, photoreceptor; Rh, rhabdom; SPC, secondary pigment cell; T, tracheole. Scale bars = 1 μm.

At the depth of roughly 68–123 μm the rhabdomeres of seven photoreceptors (R1–R7) form a fused rhabdom with cross-section 3.0 μm^2^ (Fig 5A). At roughly 88 μm depth from the corneal external surface, R7 rapidly diminishes in size and becomes much smaller than other photoreceptors (Fig 5B). The eighth photoreceptor (R8) occurs between R6 and R7 and contributes its rhabdomere to the rhabdom at the depth of approximately 123 μm (Fig 5C), where R7 moves toward the periphery of photoreceptor cluster and finally no longer contributes its rhabdomere to the rhabdom (Fig 5D). Between approximately 123–128 μm depths the rhabdom is formed by all photoreceptors (R1–R8), measuring more than 4.6 μm^2^ in cross-section area (Fig 5C). The rhabdomeres of R7 and R8 are about 60 μm and 115 μm long, respectively. From the depth of roughly 128 μm down, the rhabdom is composed of the rhabdomeres of six (R1–R6) and one (R8) photoreceptor, measuring about 4.5 μm^2^ in cross-section area (Fig 5D).

The basal matrix between the retina and the lamina is about 4 μm thick. Eight photoreceptors of the ommatidium turn into axons, which gather into a bundle passing through a round perforation to the lamina (Fig 4D).

**Fig 6:**
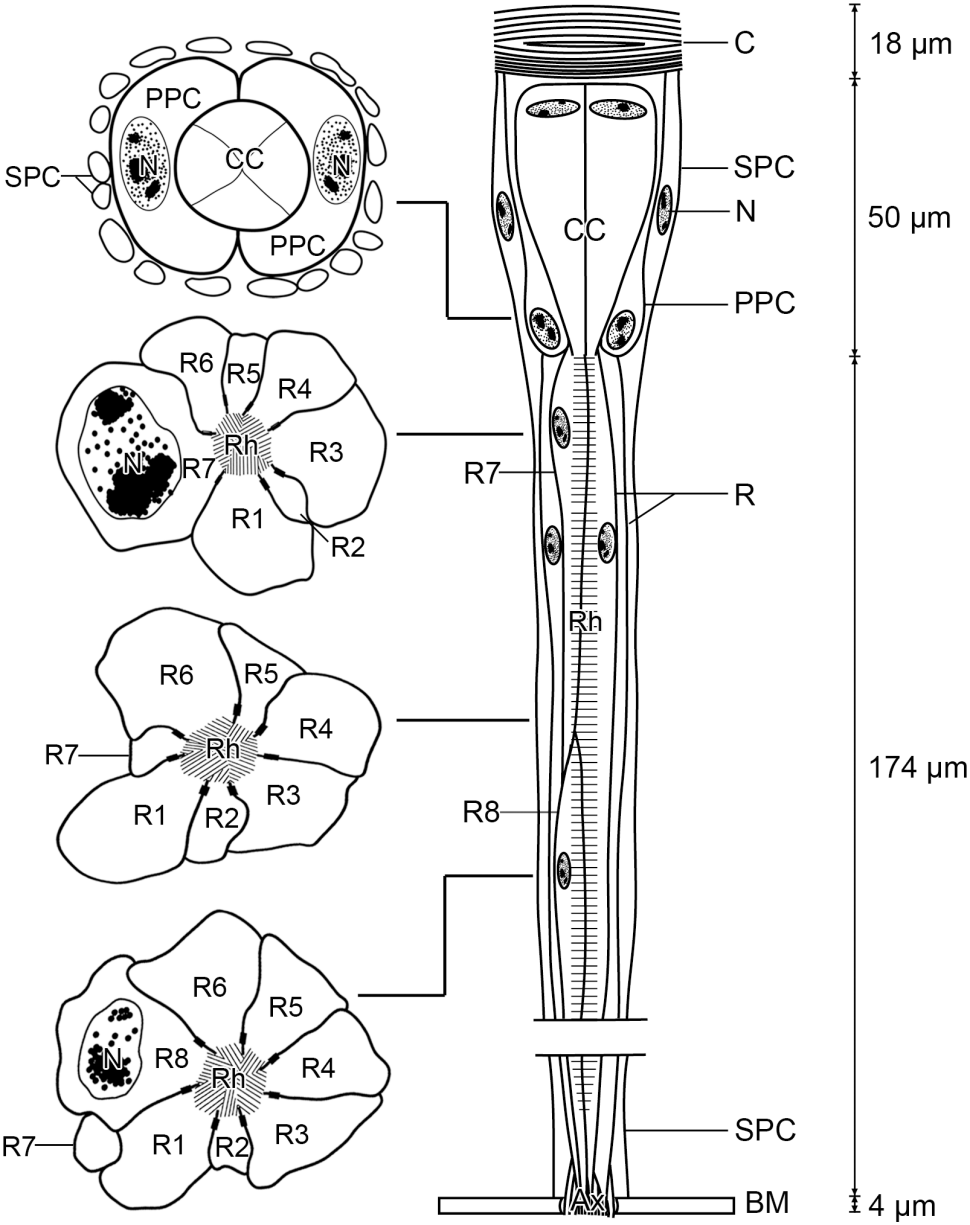
Diagram of the ommatidium of adult *Panorpa dubia* in a longitudinal and four transverse sections. Ax, axon; BM, basal matrix; C, cornea; CC, crystalline cone; N, nucleus; PPC, primary pigment cell; R, photoreceptor; Rh, rhabdom; SPC, secondary pigment cell.

Based on the above descriptions, the cellular architecture of ommatidia of adult *P*. *dubia* is diagrammatically illustrated in Fig 6.

## Discussion

The compound eyes of adult *P*. *dubia* are of the apposition type, which is related to their diurnal activity under ecological conditions [27]. The ommatidia of adult Mecoptera share a common cellular organisation of the insect compound eye, involving a cornea, a eucone crystalline cone composed of four cone cells, eight photoreceptors forming a fused rhabdom, two primary pigment cells, and a varying number of secondary pigment cells [33–35]. The ommatidial construction of Mecoptera accords with the ground plan of ommatidia of insects reviewed by Paulus [10].

The compound eyes of insects often exhibit different tiering schemes of rhabdom despite of high degree of structural conservation [1]. In all mecopteran species examined, the rhabdoms of ommatidia exhibit a slightly two-tier scheme that the rhabdomeres of R7 and the proximal R8 separately contribute to the distal and proximal part of rhabdom, whereas those of R1–R6 run along the whole length of rhabdom [33–35]. This situation is also found in the open rhabdom of some Diptera, where the central photoreceptor R7 is located directly on top of photoreceptor R8 [42,43]. The three-tier scheme holds for two butterfly families Papilionidae and Pieridae [44,45], with photoreceptors R1–R2 sensitive to UV/blue and corresponding to fly photoreceptors R7–R8, photoreceptors R3–R4 to green, and photoreceptors R5–R8 turned into red by filtering with red perirhabdomeral pigments [46,47]. In Lycaenidae and Nymphalidae, however, the tiering schemes are different [48]. We note that the electroretinogram sensitivity measurements from panorpid species [49] have shown the main sensitivity peak at 520 nm and the secondary peak at 360 nm.

The spatial resolution of insect eyes is related to the interommatidial angle [4], which varies from more than 10° in some wingless groups to 1–3° in many fast-flying predaceous insects [21]. Based on the LM section geometry, we roughly estimated that the interommatidial angle in the central part of the eye is about 6°. This is in agreement with ecological observations that most adults of Mecoptera have a relatively weak ability of flight and remain near the ground [27].

The larvae of *P*. *dubia* also have the apposition compound eyes [29]. The ommatidia of larval *P*. *dubia* almost have the same cellular components as those of their adults. Chen et al. [29] found that eight photoreceptors of larval *P*. *dubia* are arranged into four distal and four proximal photoreceptors, different from those of adults whose photoreceptors are arranged into six long, one distal, and one proximal photoreceptor. Studies on functional morphology show that the transformations of ommatidial compositions are probably a consequence of diurnal rhythms or light/dark adaptation, such as acceptance angle, cone size, and rhabdom diameter [50–53]. Similar arrangement of adult photoreceptors is also present in other adult Mecoptera [33–35] and is regarded as a general structural feature of insect ommatidia [10]. The arrangement of the photoreceptors of larval *P*. *dubia* is almost consistent with that of larval Nannochoristidae [31] and Bittacidae [32] and even adult Odonata [54], Blattaria [55], and Orthoptera [56]. Besides morphological data, the relationship study of adult and larval compound eyes needs to integrate the developmental and molecular data. The larval compound eyes degenerate during the pupal stage, whereas the adult compound eyes develop from the imaginal disc near larval eye remnants in Panorpidae [26,27]. Ando and Suzuki [36] found that the developmental processes of larval compound eyes in Panorpidae were similar to those of hemimetabolous insects. However, how the adult compound eyes develop and whether the adult and larval compound eyes use similar molecular mechanisms await further research.
